# How to Define Success in Prolactinoma Treatment—A Systematic Review and Theoretical Framework

**DOI:** 10.1210/clinem/dgaf540

**Published:** 2025-09-29

**Authors:** Victoria R van Trigt, Kevin A Huynh, Leontine E H Bakker, Iris C M Pelsma, Ingrid M Zandbergen, Amir H Zamanipoor Najafabadi, Marco J T Verstegen, Wouter R van Furth, Nienke R Biermasz

**Affiliations:** Department of Medicine, Division of Endocrinology, and Center for Endocrine Tumors Leiden, Leiden University Medical Center, Leiden 2333 ZA, The Netherlands; Department of Medicine, Division of Endocrinology, and Center for Endocrine Tumors Leiden, Leiden University Medical Center, Leiden 2333 ZA, The Netherlands; Department of Medicine, Division of Endocrinology, and Center for Endocrine Tumors Leiden, Leiden University Medical Center, Leiden 2333 ZA, The Netherlands; Department of Medicine, Division of Endocrinology, and Center for Endocrine Tumors Leiden, Leiden University Medical Center, Leiden 2333 ZA, The Netherlands; Department of Neurosurgery, Leiden University Medical Center, University Neurosurgical Center Holland, Leiden 2333 ZA, The Netherlands; Department of Ophthalmology, Leiden University Medical Center, Leiden 2333 ZA, The Netherlands; Department of Neurosurgery, Leiden University Medical Center, University Neurosurgical Center Holland, Leiden 2333 ZA, The Netherlands; Department of Neurosurgery, Leiden University Medical Center, University Neurosurgical Center Holland, Leiden 2333 ZA, The Netherlands; Department of Medicine, Division of Endocrinology, and Center for Endocrine Tumors Leiden, Leiden University Medical Center, Leiden 2333 ZA, The Netherlands

**Keywords:** prolactinoma, outcome parameters, remission, disease control, recurrence, systematic review

## Abstract

**Purpose:**

As consensus regarding outcome sets for prolactinoma treatment evaluation is lacking, this study evaluated outcome parameters reported in the literature, and objective, clinically relevant outcome sets were proposed.

**Methods:**

A systematic review of studies up to February 2, 2024. Reported biochemical and radiological parameters; clinician-reported findings; patient-reported outcomes (PROs); and definitions of disease remission, control, and recurrence were extracted and placed into a clinical context. Subsequently, objective and clinically relevant definitions of clinical outcomes were proposed based on the findings, with comprehensive outcome sets to evaluate treatment success.

**Results:**

One hundred thirty-seven articles were included. Albeit ill-defined or subjective, 23 unique prolactin parameters and 73 unique radiological parameters were reported. Seventy articles included clinician-reported findings, and none reported PROs. Ultimately, 27 unique definitions of remission, 3 unique definitions of disease control, and 20 unique definitions of recurrence were reported. We propose 2 separate definitions for biochemical and clinical remission/recurrence—either evaluating prolactin levels only or including symptomology, gonadal function, and radiology. Integrated outcome quadrants were illustrated to objectively categorize treatment success by combining achievement of treatment goals with the occurrence of adverse effects. A 3-tier outcome set based on the Value-Based Healthcare principles is provided.

**Conclusion:**

Heterogeneity in reported outcome parameters using varying definitions hampers the comparison of prolactinoma treatment outcomes. This study proposes objective, easily applicable, and clinically relevant definitions of clinical outcomes and offers a comprehensive outcome set. These parameters enable comparison of outcomes across treatment modalities and medical centers to gain insight into this rare disease and improve prolactinoma care.

Prolactinomas are generally treated with dopamine agonists (DAs), resulting in biochemical normalization in approximately 90% of patients and persistent normoprolactinemia in 16% to 21% of patients after DA withdrawal (1). An alternative first-line treatment is surgery, which results in normoprolactinemia in 80% to 92% of patients (1-3) and recurrence of hyperprolactinemia in 10% and 25% of micro- and macroprolactinomas, respectively (4).

Assessment of treatment success is more complex than solely evaluating prolactin levels and preferably includes appraisal of side effects, complications, health-related quality of life (HR-QoL), and personal treatment goals (eg, fertility). Disease heterogeneity necessitates a nuanced understanding of pathophysiology and clinical features to set patient-centered goals and interpret outcomes, especially when comparing results between treatment modalities.

Scientific interest in prolactinomas and treatment outcomes has steadily increased over the past decades. In research, a range of parameters are being used, including biochemical, radiological, and clinician-reported outcomes. Although patient-reported outcomes (PROs) are important, their role in outcome evaluations remains to be elucidated. The Value-Based Healthcare (VBHC) framework by Porter et al provides guidance on organizing outcomes in 3 tiers (4). However, this framework of standardized outcome sets has not been established for prolactinomas, leading to limited comparability between studies.

Clearly defined outcome parameters are a prerequisite for prospective registries and trials, care evaluation in pituitary centers of excellence (PTCOEs), and dedicated shared decision-making. Therefore, this study discusses clinical considerations regarding outcome parameters for prolactinomas, systematically reviews outcome measures reported in the literature, and provides suggestions for well-defined, clinically relevant outcome sets to use in clinical practice and research.

## Methods

### Study Design

This study is composed of 3 parts:

1. Clinical considerations regarding interpretation of relevant outcome parameters for prolactinomas (ie, prolactin levels, hypopituitarism and secondary hypogonadism, radiology, clinician-reported findings, and PROs, and a systematic review of their use in the literature.
2. Systematic review of reported definitions of *remission*, *disease control*, and *recurrence* and suggestions for objective, clinically relevant definitions based on considerations described in (1).
3. Recommendations for comprehensive outcome sets to evaluate prolactinoma treatment.

### Data Sources and Search

The studies included in this systematic review were partly derived from a previous systematic review and meta-analysis performed by our group, which included studies up to April 13, 2017 (2). On February 2, 2024, an update of this search strategy was performed. As shown in Table S1 (5), the strategy included 3 terms: (1) patient population, (2) treatment, and (3) outcomes (2). Seven digital libraries were searched (Academic Search Premier, Cochrane Library, Embase, Emcare, PsycInfo, PubMed, and Web of Science).

### Study Selection

Studies were selected by 2 independent researchers (K.A.H. and V.R.v.T.) following title, abstract, and full-text screening. Disagreements were resolved by discussion and consensus, and, in the case of persistent disagreement, the majority vote was chosen by consultation of a third researcher (I.C.M.P.). Randomized controlled trials, cohort studies, and cross-sectional studies reporting on outcomes of medical treatment and transsphenoidal surgery of radiologically confirmed prolactinomas were included if they reported 1 of the following outcomes: disease remission after DA or transsphenoidal surgery, disease control on DA, disease recurrence, or HR-QoL. The following studies were excluded: reporting primarily on patients <16 years old, fewer than 10 patients, or without original data. When cohorts overlapped, the largest cohort was included.

### Data Extraction

The following data were extracted independently by K.A.H. and V.R.v.T.: publication year, study design; duration of follow-up; DA withdrawal criteria; and definitions of disease control, recurrence, and remission. Moreover, all reported definitions describing prolactinoma outcomes, including biochemical, radiological, and clinical (clinician-reported findings and PROs) were collected. In this systematic review, the physicians’ interpretations of medical history, physical examination, and (ophthalmological) function tests were considered clinician-reported findings. Normoprolactinemia was defined as prolactin within the laboratory-specific reference range standardized for sex and menopausal status. Details concerning the extracted variables are summarized in Table S2 (5).

## Results

### Included Studies (Fig. 1)

A total of 1703 unique studies were identified, of which 1362 were excluded based on title and abstract. Full-text screening of 341 articles was performed, after which 137 studies were included in this review. In total, 66 studies reported on medical outcomes, 54 studies reported on surgical outcomes, and 17 studies reported on both. All extracted data are shown in Table S3 (5).

**Figure 1.**
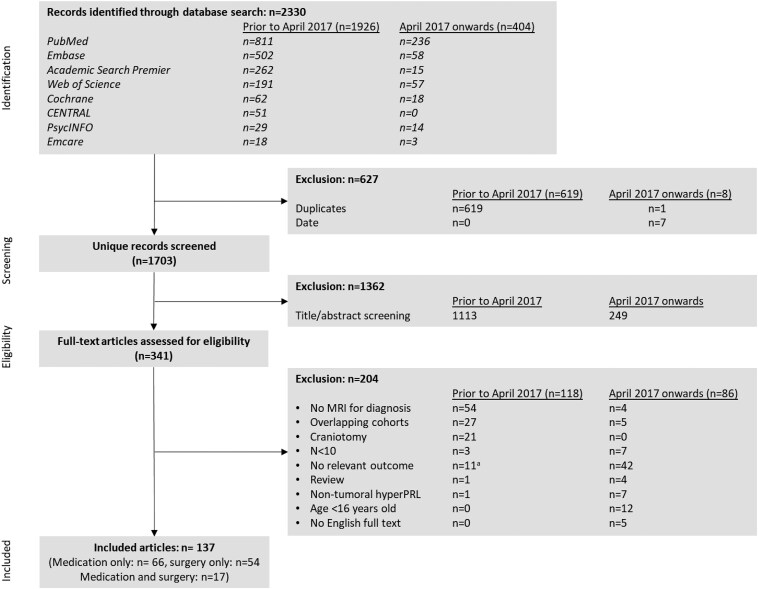
Flow chart of screening and inclusion. ^a^Four studies that were included in the previous systematic review were excluded from the current study, as they only reported on side effects or costs without evaluating treatment success. Abbreviation: HyperPRL, hyperprolactinemia.

## Evaluation of Outcome Parameters Described in the Literature

### Prolactin Levels

#### Clinical considerations and interpretation

Serum prolactin is determined using automated immunometric immunoassays, in which the prolactin protein reacts with immobilized capture antibodies and labeled antibodies for detection. The signal generated after washing is proportional to the sample's prolactin concentration (6). Manufacturers provide assay-specific reference ranges for men, pre- and postmenopausal women, and sometimes for pregnant women. The expected reference ranges supplied by the manufacturer are established by testing samples from approximately 100 to 200 healthy individuals and determination of the central 95% of values. These are to be verified by laboratories for their own population (6). Although reference ranges vary between laboratories (7, 8), reporting the upper limit of normal (ULN) facilitates interpretation across centers. Serum prolactin is a sensitive and objective measure of disease activity without necessitating dynamic testing. However, physiological variations of individual prolactin setpoints not corresponding with established reference ranges may occur.

When evaluating prolactinoma treatment outcomes, it is important to consider that various other conditions may induce (additional) hyperprolactinemia (generally <5xULN), potentially interfering with outcome interpretations (eg, liver cirrhosis, renal insufficiency, primary hypothyroidism, or stalk compression). Moreover, antipsychotics, antidepressants, anti-emetics, protease inhibitors, and opiates may induce hyperprolactinemia (9). Furthermore, physiological alterations, caused by the ovulatory and luteal phase of the menstrual cycle, pregnancy, stress, exercise, and high-protein meals, cause hyperprolactinemia of varying severity (10). Repeated cannulated prolactin sampling may rule out some of these causes of hyperprolactinemia at initial diagnosis, but also during evaluation of treatment success—particularly in cases with recovery of the gonadal axis in the absence of complete normalization of prolactin levels.

To correctly interpret prolactin levels, clinical manifestations and radiology should be taken into account. For instance, macroprolactinemia, caused by antiprolactin antibodies (mostly IgG) bound to prolactin, can lead to biologically inactive hyperprolactinemia detected by the assay (10). Prolactin recovery <40% after polyethylene glycol precipitation confirms macroprolactinemia (10). Macroprolactinemia should be ruled out in patients with asymptomatic hyperprolactinemia (<200 ng/mL) during the diagnostic phase (11), and if a combination of macroprolactinemia and monomeric prolactinoma-related hyperprolactinemia is present, this should be considered during evaluation of disease activity. Furthermore, although rare in modern assays, high-dose hook effects lead to falsely normalized or slightly elevated prolactin measurements in extreme hyperprolactinemia by saturating assay antibodies (12). As prolactin usually positively correlates with tumor size, samples in patients with giant adenomas and typical prolactinoma symptoms should be diluted (11). Thus, prolactin is an essential parameter in evaluating treatment success that should be interpreted considering clinical findings and radiology.

#### Reported prolactin parameters [Table S4 (5)]

Prolactin levels were reported in 136 out of 137 studies. Twenty-three unique prolactin parameters were used: prolactin normalization (96 studies), absolute (nadir) posttreatment prolactin (78 studies), categorical parameters (eg, prolactin decrease yes/no) (3 studies), percentages of change (4 studies), and undefined outcomes such as “prolactin near ULN” (1 study) and “prolactin <10 ng/mL” without providing the ULN (1 study each).

Two studies reported using cannulated, unstressed prolactin levels (13, 14), whereas the other studies did not report on the method of prolactin measurement. Two studies reported average prolactin levels of multiple measurements (15, 16). None of the studies reported on the interpretation of prolactin levels in case of concomitant use of prolactin-elevating medication or pregnancy during follow-up.

#### Prolactin levels in the reported definitions of disease remission [Fig. 2, Table S5 (5)]

Prolactin levels were included in all but 1 definition of remission (17). Remission entailed normalization of prolactin below ULN in most studies, with 2 studies aiming for other cut-off values (18, 19). Five studies mentioned a cut-off value without stating the ULN (20-24), and 1 study stated the unspecified aim of achieving “healthy prolactin levels” (25). One definition of remission allowed either normalization of prolactin or reduction of >95% of baseline prolactin (26), and another allowed asymptomatic prolactin values up to 1.5xULN (27). One study reported separate definitions for biochemical and clinical remission, allowing asymptomatic prolactin levels above the ULN for “clinical remission” if gonadal function was restored (28).

**Figure 2.**
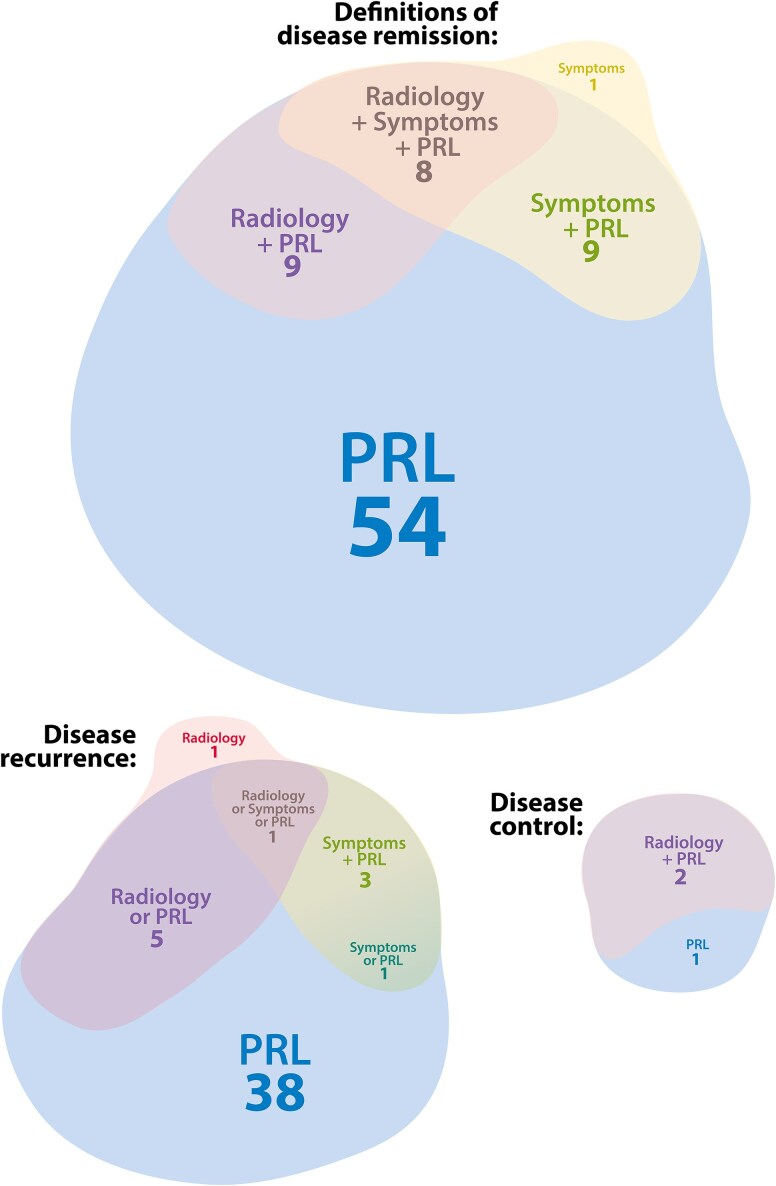
Visual representation of parameters used in the definitions of *disease remission*, *disease recurrence,* and *disease control* as reported in literature. The number of studies using the specific parameters in their definition is provided. Sixty-three studies reported a total of 81 definitions of remission, among which 27 were unique definitions. Forty-six studies reported 49 definitions of disease recurrence, among which 20 unique definitions, and 3 studies reported 4 unique definitions of disease control. “Symptoms” concern clinician-reported symptoms. “Radiology” is magnetic resonance imaging of the pituitary. Abbreviation: PRL, serum prolactin.

### Gonadal Status

#### Clinical considerations

Prolactin suppresses the gonadal axis by direct suppression of GnRH and indirectly through kisspeptin (29, 30). Therefore, restoration of the gonadal axis is an indicator of treatment success. In females, secondary hypogonadism is characterized by low estradiol levels combined with inadequately low to normal LH and FSH leading to anovulatory menstrual cycles (31). Gonadotrophins should be measured in the morning and be interpreted considering the patient's age and phase of the menstrual cycle (31, 32). In males, low serum testosterone levels and/or reduced spermatogenesis with inadequately low to normal gonadotrophins are indicative of secondary hypogonadism (33). Serum testosterone is a marker for androgen status and should be measured in the absence of acute illness before 10 Am after an overnight fast to account for its diurnal rhythm and food- and illness-induced suppression (34-36). Approximately 2% to 4% of testosterone circulates in unbound bioactive form, whereas approximately 44% is tightly bound to SHBG and 50% to 54% is loosely bound to albumin (34, 36, 37). Therefore, measurement of free testosterone is advised in patients with total testosterone levels near the lower limit of normal or with suspected albumin or SHBG abnormalities. Taking these factors into account, the clinical diagnosis of female and male secondary hypogonadism can be made. However, in clinical practice, the interpretation of female gonadotropins might be complicated, as the phase of the cycle is not always known. Moreover, other causes of acquired secondary hypogonadism, including mass effect- or trauma-related pituitary or hypothalamic damage; ischemic, inflammatory, or infectious diseases; metabolic disturbances; drug-induced hypogonadism; and functional hypothalamic dysfunction, should be considered (31, 32, 38). Thus, restoration of gonadal function [ie, restoration of the menstrual cycle with persisting estradiol levels >200 pmol/L or normalization of morning fasting free testosterone levels (31, 33)] is a good indicator for prolactinoma disease control, with pregnancy being proof of eugonadism. However, persistent hypogonadism should not always be interpreted as treatment failure because other factors may contribute.

#### Reported parameters concerning hypogonadism [Table S4 (5)]

Twenty-six studies reported on the improvement of hypopituitarism (including hypogonadism) after treatment. Nineteen studies reported specifically on the improvement of hypogonadism, of which only 2 stated a definition. Andereggen et al defined hypogonadism as “normal-low levels of gonadotropins in parallel with low estradiol levels,” and Kabootari et al defined central hypogonadism as “amenorrhea for >3 months in women, and sexual dysfunction in men with low levels of gonadal hormones and normal to low levels of LH and FSH” (39).

#### Gonadal status in the reported definitions of disease remission [Fig. 2, Table S5 (5)]

Two studies included the gonadal status or the menstrual cycle in their definition of remission, with 1 defining remission as “reoccurrence of normal menstrual cycle and PRL < 10 ng/mL that could be normally stimulated ≥2.5xULN by thyrotropin-releasing hormone and metoclopramide” (21). The other study defined clinical remission as “restoration of the gonadal function and resolution of complaints without prolactin normalization” (28).

### Radiological Parameters

#### Clinical considerations

Magnetic resonance imaging (MRI) is the gold standard for radiological prolactinoma diagnosis, and it provides information about the prolactinoma's relationship with critical structures. Tumor shrinkage is an objective measure of response to medical treatment, and knowing there is no clear remnant on MRI may relieve patients’ stress. Moreover, the absence of residual tumor mass after DA withdrawal is positively correlated with the probability of persistent normoprolactinemia (11, 40, 41), and consecutive posttreatment imaging can identify subtle growth of a remnant and determine the feasibility of (re)resection. However, prolactin levels are superior to evaluate total resection, as the interpretation of imaging after surgery is hampered by postoperative hemorrhage and fluid collections early after surgery, and complexity of discriminating between resection cavity and scar tissue at later timepoints (42). Moreover, MRIs are not suitable for repeated screening for recurrent disease, due to insufficient sensitivity for small remnants and excessive costs. Thus, imaging offers prognostic information about the durability of remission and enables evaluation of decompression yet has limited value in the evaluation of disease remission.

#### Reported radiological parameters [Table S6 (5)]

Ninety-six studies reported on radiological outcomes. In total, 73 unique parameters were used. (Gross) total resection (27 studies) and complete tumor disappearance after DA treatment (27 studies) were reported most frequently. Five unique parameters concerning volume or tumor mass were reported (23 studies), and 7 concerned tumor diameter (24 studies).

Fifteen different percentages of tumor volume/diameter reduction were reported. Eleven subjective undefined measures were reported, such as “subtotal resection’ (7 studies) and “significant debulking/tumor shrinkage” or “partial removal” (1 study each).

One study reported on the lesion's intensity on MRI and the time to appearance of low signal intensity (months) (43), and 1 reported on cystic degeneration (44).

#### Radiological parameters in the reported definitions of remission [Fig. 2, Table S5 (5)]

Thirteen studies included radiological parameters in their definition of remission [medical n = 5 (45-49), surgical n = 10 (17, 22, 27, 48-54)]. Most studies aimed for the absence of a remnant on MRI. However, 1 study aimed for stable tumor size after DA withdrawal (46), and another study on surgical remission aimed for >50% tumor reduction on MRI 3 months postsurgery (22).

### Clinician-reported Findings

#### Clinical considerations

Clinician-reported findings offer indirect insight into patients’ well-being and HR-QoL, being derived from the interpretation of clinical signs and symptoms. Physicians who know their patient well can identify overlooked symptoms and assess their severity objectively due to their clinical experience. Contrary to patient-reported outcome measures (PROMs), clinical history taking offers the flexibility to rephrase questions to ensure the patient’s understanding and to focus on personalized topics. Moreover, clinician-reported findings are particularly relevant in patients unable to complete PROMs. Unfortunately, they have been shown to correlate poorly with PROMs (48-50). Moreover, their use in research is hampered by incomplete and unstandardized reporting in (electronic) health records. Hence, clinician-reported outcomes complement PROMs without replacing them.

#### Reported clinician-reported findings and visual measurements [Table S7 (5)]

Seventy studies reported clinician-reported findings. Visual symptoms were most frequently reported, with 28 studies reporting on visual field defects, 3 studies on visual acuity, and 16 studies on unspecified “visual symptoms.” Galactorrhea, headaches, and subfertility were reported by 13, 11, and 4 studies, respectively. Only 2 studies reported mood disturbances. Eleven studies reported “symptoms” without further specification.

#### Clinician-reported findings in the reported definitions of remission [Fig. 2, Table S5 (5)]

Sixteen studies included clinician-reported findings concerning symptomatology in their definition of remission (17, 21, 25, 27, 28, 49, 51-60). Fifteen studies aimed for complete resolution of symptoms, without specifying which symptoms, and 1 study aimed for “evidence of clinical remission” without stating its definition (60).

### Patient-reported Outcomes

#### Clinical considerations

PROMs can be either generic, such as the Short Form 36 and EuroQoL 5D, or pituitary-specific, such as the Leiden Bothers and Needs Pituitary. The Leiden Bothers and Needs Pituitary is the only validated questionnaire for pituitary adenomas, measuring disease burden (bothers) and needs for attention of the treating physician (46). Generic PROMs, widely used in the literature, enable comparison between studies and diseases. However, they may lack the sensitivity to detect prolactinoma-specific changes in HR-QoL (47). Therefore, using a combination of both is advised (7).

PROMs aim to assess the patient’s perspective on disease, treatment, and HR-QoL, without subjective interpretation by healthcare providers. Moreover, the patient might feel freer to report sensitive topics that are difficult to discuss in person. However, one must realize that PROMs can merely approximate true HR-QoL. It remains unclear to what extent a single PROM accurately reflects the multifaceted and dynamic nature of HR-QoL. Moreover, PROMs have a few inherent limitations that should be considered. Firstly, PROMs are subject to varying interpretation, based on age, gender, cultural background, reading ability, understanding of the language, and phrasing of questions—with most not being validated in prolactinomas. Secondly, PROMs are composed of a fixed set of questions that are not tailored to the individual, potentially causing confusion when questions do not align with the patient's situation. Thirdly, responses may be influenced by comorbidity, mood, life events, and recall bias. Although it is valuable to gain insight into the patient's overall well-being, this complicates the use of PROMS in treatment evaluations. Fourthly, the time burden of completing PROMs may decrease response rate, hampering the validity of outcomes due to selective nonresponse. Lastly, implementation in clinical practice may be challenging and time-consuming. Thus, PROMs are essential to gain insight into HR-QoL, yet interpretation of the results and implementation in clinical practice are complex.

#### Reported patient-reported outcomes

No studies reported on PROMS.

## Proposed Definitions (Table 1)

Definitions of remission, disease control, and recurrence as reported in literature are evaluated next, and propositions for clinically relevant definitions are made.

**Table 1.** Proposed definitions of clinical outcomes

Clinical outcome		Definition
Disease remission	Biochemical	Normoprolactinemia*^a,b^* >3 months after DA withdrawal or >6 weeks postsurgery while off DA for >3 months
	Clinical	Prolactin >1xULN or with uninterpretable prolactin levels*^c^* with resolution of typical prolactinoma-related symptoms (ie, galactorrhea, loss of libido, subfertility, menstrual cycle disturbances, or erectile dysfunction),*^d^* and recovery of gonadal function without a certain remnant on conventional MRI and without treatment indication
	Radiological	No adenoma remnant on conventional MRI
Disease control	Prolactinoma without mass effects	Normoprolactinemia*^a^* during DA treatment without typical prolactinoma-related symptoms (see above) and with stable or decreased tumor size*^e^* in all dimensions on conventional MRI
	Prolactinoma causing mass effects	Normoprolactinemia*^a^* during DA treatment without typical prolactinoma-related symptoms (see above) and with radiologically and clinically*^f^* confirmed resolution of compression and without size increase in any dimension on conventional MRI
	Radiological	Stable or decreasing adenoma size on conventional MRI
Disease recurrence	Biochemical	Hyperprolactinemia (>1.0xULN) measured at least twice*^g^* after initial biochemical remission
	Clinical Radiological	Recurrence of prolactinoma-related*^h^* symptoms that had receded after initial biochemical or clinical disease remission
		Reappearance of an adenoma on conventional MRI after radiological remission
Hypogonadism	Female	Oligomenorrhea or amenorrhea with persisting levels of serum estradiol below 200 pmol/L
	Male	Morning fasting serum testosterone levels below the lower limit of normal after correction for albumin and sex hormone-binding protein

Abbreviations: DA, dopamine agonist; MRI, magnetic resonance imaging; xULN, times upper limit of normal.

^*a*^Normoprolactinemia is defined as prolactin within the laboratory-specific upper limit of normal for sex and menopausal status.

^*b*^In case of mild hyperprolactinemia (<5xULN), a second unstressed, fasting cannulated measurement during the early follicular phase of the menstrual cycle should be performed if physiological prolactin elevation is suspected.

^*c*^Due to pregnancy, lactation, or prolactin-elevating medication.

^*d*^Symptoms should preferably be patient-reported. Clinician-reported symptoms may be used if patient-reported data is not available.

^*e*^Either tumor volume or the largest tumor diameter.

^*f*^Excluding permanent visual deficits due to previous compression, as evaluated by the multidisciplinary team of a pituitary center of excellence.

^*g*^The second measurement should be an unstressed, fasting, cannulated measurement during the early follicular phase of the menstrual cycle if physiological prolactin elevation is suspected.

^*h*^Symptoms that the patient recognizes from the initial prolactinoma diagnosis.

### Disease Remission [Fig. 2, Table S5 (5)]

As reported earlier, published definitions of remission were heterogeneous, consisting of varying combinations of parameters. Sixty-three studies reported 81 definitions of remission, among which 27 were unique definitions. The most commonly reported definition was prolactin normalization (n = 42), of which 17 definitions required the patients to be withdrawn from DA treatment; 2 definitions allowed patients to be on DA treatment; and 23 definitions did not specify DA treatment status. Timing of measurements varied greatly, with most studies reporting remission at last follow-up.

#### Biochemical remission

According to the *Merriam-Webster Dictionary*, the medical term *remission* is defined as “a state or period during which the symptoms of a disease are abated” (61). As illustrated by many variations throughout literature, this definition is difficult to apply to prolactinoma, with symptoms being nonspecific (eg, headaches and psychological and cognitive complaints) and potentially caused by unrelated pathology. Moreover, cognitive and psychological complaints may persist for years after biochemical normalization (62). Requiring resolution of all potentially prolactinoma-related symptoms may cause overtreatment and underestimation of true remission rates.

Prolactin normalization is a more reliable marker for remission, as used in most studies. Complete normalization (<1.0xULN) is desirable, as recurrence rates are lowest in patients in whom normoprolactinemia has been achieved (3). Although radiological tumor disappearance was a common criterion for remission, we propose excluding radiological parameters from the definition of biochemical remission (due to insufficient sensitivity). Thus, we propose to define *biochemical remission* as prolactin levels within the laboratory-specific reference range (<1.0xULN) > 6 weeks postsurgery and >3 months after DA withdrawal, due to the long half-life of cabergoline.

#### Clinical remission

Biochemical remission does not imply the absence of symptoms or restoration of HR-QoL, as recovery is a process and symptoms may (partly) persist. By contrast, symptoms may recede and the gonadal axis may recover despite mild persisting hyperprolactinemia due to issues described earlier. The absence of biochemical remission should, therefore, not always be interpreted as treatment failure.

The term *clinical remission* is suitable in the following specific clinical scenarios: prolactin levels >1xULN, or uninterpretable prolactin levels (due to pregnancy, lactation, or comedication), without typical prolactinoma-related symptoms [ie, galactorrhea, loss of libido, subfertility, menstrual cycle disturbances, or erectile dysfunction (11)], with recovery of gonadal function, and without a certain remnant on MRI or treatment indication. The prolactinoma-related symptoms should preferably be patient-reported but may also be clinician-reported if not available.

Although optimizing HR-QoL is the most important treatment goal for patients with a prolactinoma, including HR-QoL as a factor in the definition of clinical remission is not appropriate due to several reasons. Firstly, remission status is merely 1 of the many factors that affect HR-QoL (63). Due to its multifactorial determinants, HR-QoL is highly variable, and quantifying HR-QoL is inherently complex. Second, it remains uncertain to what extent HR-QoL can be accurately captured by PROMS, and third, there are no reference ranges, especially no age- and sex-dependent ones. Therefore, including HR-QoL or using PROMS as a proxy for HR-QoL in the definition of clinical remission is not feasible. Alternatively, by incorporating objective measures such as symptomatology, gonadal status, and radiological outcomes, we believe that achieving clinical remission may contribute to improved HR-QoL.

Differentiating between biochemical and clinical remission is relevant for 2 reasons: (1) to classify patients with a clinically satisfactory result not adhering to the strict biochemical criteria and (2) to enable a more holistic treatment evaluation. While biochemical remission is the preferred outcome in research, clinical remission is the most relevant outcome in clinical practice. Both are meaningful and have their merits.

#### Radiological remission

As mentioned, radiological remission (ie, the absence of an adenoma remnant on conventional MRI) is less informative regarding the persistence of active disease than biochemical or clinical remission status, as MRI has limited sensitivity for small (postoperative) remnants. On the other hand, the presence of an adenoma remnant on conventional MRI does have prognostic value and should be reported in combination with biochemical/clinical remission status.

#### Evaluating remission in pregnant patients

Patients becoming pregnant during follow-up pose a challenge in research, due to the physiological rise of prolactin levels (10). Ultimately, conception is an excellent outcome for patients wishing to conceive, demonstrating restoration of gonadal function. Patients who are pregnant at the time of outcome assessment can, therefore, be classified as being in *clinical remission* if they conceived without the use of DAs or medical assistance. Biochemical remission should be assessed 6 weeks after cessation of breastfeeding (11, 64-68).

#### Disease control [Table S8 (5)]

Three studies reported 4 unique definitions of disease control. One study differentiated between complete (ie, prolactin normalization) and partial disease control (ie, prolactin reduction to <3xULN without normalization while using DAs) (69). The other studies required prolactin normalization with “stable neuroimaging” during DA treatment (49) or with >50% tumor reduction without specifying DA treatment status (48). Two studies reported disease control at last follow-up (49, 69), and 1 study did not report the timing of measurement (48).

Disease control implies the disease sequelae are suppressed by medical treatment, without full resolution of the disease itself. In accordance with most studies, we propose to define *disease control* as follows: prolactin normalization while using DAs without typical prolactinoma-related symptoms [ie, galactorrhea, loss of libido, subfertility, menstrual cycle disturbances, or erectile dysfunction (11)]. For noncompressive prolactinomas, adenoma diameter/volume on MRI should be stable or reduced. In patients with compressive prolactinomas, tumor diameter/volume should be reduced until compression is alleviated (radiologically and clinically). Prolactin levels should not be medically lowered below the laboratory-specific lower limit of normal, because hypoprolactinemia is associated with impaired metabolic health (70, 71), sexual dysfunction, and depressive symptoms (72, 73). Conversely, temporary hypoprolactinemia directly postoperative is desirable, as lower postoperative prolactin levels are predictive of long-term cure (74) and typically return to normoprolactinemic levels in the following weeks to months.

#### Radiological control

Radiological control is defined as the presence of a stable or decreasing adenoma diameter in all dimensions on conventional MRI, irrespective of prolactin levels.

#### Discordant responses to treatment

DAs effectively induce normoprolactinemia and tumor shrinkage in most cases (1). Prolactin levels and tumor size decrease most rapidly during the first 6 months of treatment (64), and the decline of prolactin levels typically precedes tumor shrinkage (65). More rarely, treatment responses are discordant, with volume increase despite a marked drop in prolactin levels or tumor shrinkage without declining prolactin levels. Interestingly, none of the studies included in this systematic review described discordant responses. Our proposed definitions may aid in correctly classifying these patients.

Case reports describing tumor enlargement despite declining prolactin levels concern macro- and giant prolactinomas treated with either bromocriptine or cabergoline (75-77). Potential explanations include noncompliance with treatment or a different etiology, ie, nonfunctioning adenomas with stalk compression, asymptomatic apoplexies, or cystic degeneration (66, 75, 76, 78). In some cases, perceived adenoma enlargement may be explained by the improved sensitivity of computed tomography (CT) scans rather than adenoma enlargement—as most reports describing discordant adenoma growth concern early cases in which CT scans were used instead of MRI (66, 76). Alternatively, tumor shrinkage with worsening hyperprolactinemia has been described (67). This phenomenon may be caused by physiological or drug-induced hyperprolactinemia (as described earlier) or, more rarely, by distant metastases as seen in pituitary carcinomas (11).

Thus, discordant treatment responses should prompt further investigation to confirm or exclude alternative etiologies, even though the underlying mechanisms may not always be identifiable. When categorizing patients with discordant responses, those who are normoprolactinemic with stable-sized, noncompressive adenoma may be considered to have controlled disease. Conversely, tumor growth is an indicator of inadequate disease control. Patients with persisting functional hyperprolactinemia should be classified as uncontrolled, irrespective of tumor shrinkage.

### Disease Recurrence [Table S9 (5)]

Forty-six studies reported 20 unique definitions of disease recurrence. Most definitions required elevation of prolactin above ULN irrespective of radiological findings or symptoms, without the need for repeat measurements (n = 38). Two studies applied alternative prolactin cut-offs of >2xULN (79) and >30 ng/mL without reporting reference values (13). Three studies required recurrence of hyperprolactinemia and clinician-reported symptoms (27, 54, 80), and 5 studies defined recurrence as either hyperprolactinemia or tumor regrowth on MRI (17, 22, 48, 81, 82). One study required either radiological tumor regrowth or symptoms (51), and 1 study required either hyperprolactinemia, adenoma regrowth, or recurrence of symptoms (83). One study differentiated between early and late recurrence (being either ≤3 months or >3 months postsurgery) (57), and 2 studies differentiated between “biologic” or “general recurrence” (ie, hyperprolactinemia) and “radiological recurrence” (ie, tumor regrowth) (56, 84). Most studies evaluated recurrence throughout follow-up, without predefined timing of evaluation.

We propose to differentiate between *biochemical* and *clinical recurrence*, as not all biochemical recurrences necessitate treatment, whereas clinical recurrences do. We propose to define *biochemical recurrence* as confirmed serum prolactin elevation (>1xULN) in 2 separate measurements after initial biochemical remission with exclusion of physiological and drug-induced hyperprolactinemia. The second measurement should be an unstressed, cannulated measurement during the early follicular phase of the menstrual cycle if physiological hyperprolactinemia is suspected (as mentioned above). We propose to define *clinical recurrence* as recurrence of prolactinoma-related symptoms that had receded after initial biochemical or clinical disease remission. Routine radiological surveillance is not advised after achievement of remission. In cases with increasing prolactin levels (not yet reaching the ULN), *radiological recurrence* (ie, reappearance of an adenoma on conventional MRI after initial radiological remission) may provide an additional argument for the presence of active disease. In the absence of increasing prolactin levels, one should consider alternative etiologies such as a nonfunctioning adenoma or postoperative changes.

## Clinically Relevant Outcome Sets

An objective, easily applicable, and clinically relevant outcome set incorporating personalized treatment goals is discussed next.

### Treatment Goals

Establishing treatment goals prior to treatment initiation is essential to determine relevant outcome parameters, as these may vary throughout the heterogeneous patient population. Patients can be grossly categorized into 2 main groups according to treatment goals. The first group concerns patients with noninvasive micro- and macroprolactinoma who generally have been diagnosed due to galactorrhea or hypogonadal symptoms. Optic chiasm compression may be present in some compressive yet noninvasive macroadenomas. Achieving sustained normoprolactinemia (ie, biochemical remission) is the primary treatment goal, with decompression of the optic chiasm potentially being a second goal. In specific cases in which normoprolactinemia cannot be achieved, for instance due to DA resistance or intolerance and a preference to withhold from surgery, one may opt for hormonal replacement therapy without normoprolactinemia.

The second group consists of patients with larger invasive and compressive macro- and giant prolactinomas. Besides hyperprolactinemia-related symptoms, well-being is impaired due to mass effects, and DA-free normoprolactinemia is usually not feasible. The primary treatment goal is alleviation of mass-related symptoms through mass reduction, and the second goal is lowering or normalizing prolactin levels, with either natural restoration of gonadal function or gonadal replacement therapy.

Additionally, there is a subgroup of patients treated for subfertility. Although these patients may experience hyperprolactinemic and mass-induced symptoms, an important goal of treatment is conception of a healthy child. This personal goal should be combined with the goals described earlier.

### Categorizing Treatment Outcomes: Integrated Outcome Quadrants (Fig. 3)

Integrated outcome quadrants (IOQs) can objectively categorize treatment outcomes into 4 groups based on achievement of treatment goals and adverse effects. This concept was introduced to evaluate surgical treatment in pituitary adenomas (85) and is also applicable to medical treatment. IOQs incorporate individualized treatment goals, enabling objective comparison of results between PTCOEs and treatment modalities. The IOQ for biochemical remission is useful after DA withdrawal or surgery aiming for total resection, whereas the IOQ for disease control can be used for patients using DAs. The IOQs evaluating mass effects and fertility are useful for patients with compressive prolactinomas and patients treated for subfertility, respectively [Fig. S1 (5)].

**Figure 3.**
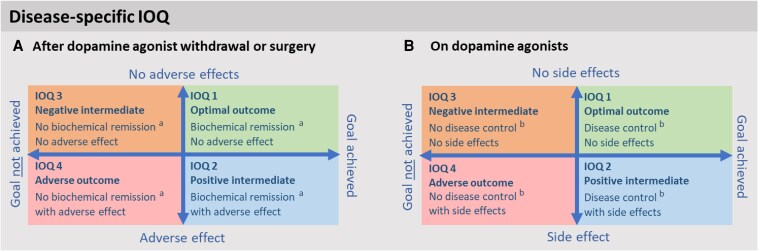
Integrated outcome quadrants for evaluating treatment success (A) after dopamine agonist withdrawal or surgery and (B) while using dopamine agonists. ^a^Biochemical remission is defined as normalization of serum prolactin (within the upper limit of normal) without the use of dopamine agonists. ^b^Disease control is defined as normalization of serum prolactin and resolution of typical prolactinoma-related symptoms (ie, galactorrhea, loss of libido, subfertility, menstrual cycle disturbances, or erectile dysfunction—Pituitary Society consensus statement, 2023) with resolution of compression and without size increase in any dimension for patients with compressive prolactinoma or with stable or decreased size in patients with noncompressive prolactinoma. Abbreviation: IOQ, integrated outcome quadrant.

To enable holistic outcome evaluations in clinical practice, a patient-centered IOQ may be used next to the disease/symptom-specific IOQs when applicable. This IOQ uses a combination of outcome parameters consistent with the term *clinical remission*: recovery of hypogonadism, resolution of typical prolactinoma symptoms [galactorrhea, loss of libido, subfertility, menstrual cycle abnormality, and erectile dysfunction (11)], and no certain lesion on MRI or treatment indication [Fig. S1 (5)].

### Outcome Sets for Evaluation of Prolactinoma Treatment (Fig. 4)

The 3-tier hierarchy by Porter et al offers a comprehensive framework based on the VBHC principles to report outcomes during 3 phases of the care cycle, aiming to improve quality of care and enhance cost-efficacy by focusing on patient-relevant outcomes (4).

**Figure 4.**
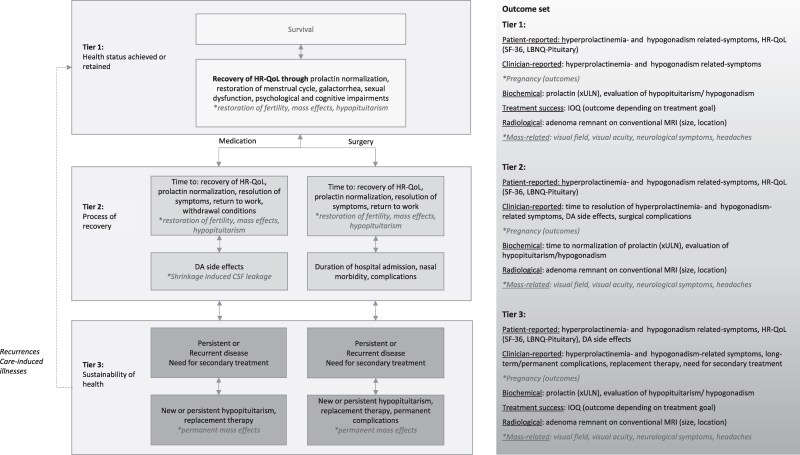
Three-tier Value Based Healthcare model for prolactinomas. *When applicable. Abbreviations: CSF, cerebrospinal fluid; DA, dopamine agonist; HR-QoL, health-related quality of life; IOQ, integrated outcome quadrant; LBNQ-Pituitary, Leiden Bothers and Needs Pituitary; SF-36, Short Form 36; xULN, times upper limit of normal.

The first tier focuses on the health status achieved or retained, ie, core outcomes at the endpoint of treatment: HR-QoL (measured using a generic and a disease-specific PROM), the applicable IOQ to evaluate treatment success [Fig. 3, Fig. S1 (5)], and normalization of prolactin levels with restoration of hyperprolactinemia- and hypogonadism-associated symptoms. Optional goals include restoration of mass-related symptoms and fertility. We advise evaluating treatment success after approximately 2 years for medically treated patients (after potential withdrawal attempts) or 1 year after surgery to allow restoration to endocrine equilibrium.

The second tier describes the recovery process, ie, health changes from baseline to the endpoint of treatment, including the time to recovery of HR-QoL, prolactin normalization, resolution of symptoms, and sometimes return to work. Time to achieve withdrawal conditions and DA side effects should be reported for medically treated patients, and the duration of hospitalization and complications should be reported for surgically treated patients. The second tier starts at initiation of treatment and ends approximately 2 years after medication initiation or 1-year postsurgery (as described earlier).

The third tier describes the sustainability of health, including persistent or recurrent disease and the need for secondary treatment. Relevant factors include an IOQ (long-term disease success), long-term complications, persisting or new hypopituitarism, and need for hormonal replacement. Permanent mass-induced symptoms should be evaluated after treatment of compressive prolactinomas. A suitable time to measure the third tier is approximately 5 years after treatment initiation.

## Discussion

Many, mostly subjective, prolactinoma outcome parameters were reported in literature, and definitions of clinical outcomes varied across studies. This systematic review evaluates outcome parameters and provides suggestions for clinically relevant, objective definitions. Ultimately, a standardized outcome set depending on individual treatment goals is proposed.

Standardization of outcome sets is important to compare outcomes across PTCOEs. Ideal outcome parameters should be objective, easily applicable, and clinically relevant. The definitions of *biochemical remission, disease control,* and *recurrence* as shown in Table 1 adhere to these criteria. Because mild hyperprolactinemia may persist due to physiological processes or comedication or because prolactin levels may be uninterpretable (eg, during pregnancy), we emphasize the need for a patient-centered definition to describe this group. Although the term *clinical remission* is less objective, and the possibility of a small remnant cannot be excluded, it enables a more realistic categorization of patients with a clinically satisfactory result, albeit not adhering to the strict criteria for biochemical remission. Use of these definitions in research can improve comparability of studies, thereby enhancing knowledge about prolactinoma treatment.

IOQs—integrating treatment goals and adverse effects—categorize treatment outcomes, enabling comparison of outcomes between treatments and PTCOEs irrespective of treatment goals (85). Setting individualized goals prior to treatment initiation is a prerequisite for the use of IOQs. The definitions of remission and disease control as described earlier can be used within this framework [Fig. 3, Fig. S1 (5)]. The holistic, patient-centered IOQ allows a more personal approach and may support treatment choices.

PROMs were not included in evaluations of treatment success in literature, although optimizing HR-QoL is an important goal of treatment. Intuitively, HR-QoL improves after prolactin normalization and adenoma shrinkage, because hyperprolactinemia-, hypogonadism-, and mass-related symptoms should recede; however, little is known about HR-QoL after prolactinoma treatment (86). A few aspects may prevent researchers from using PROMs. Firstly, interpretation of PROMs is complex, as outcomes are affected by many factors other than the prolactinoma itself (eg, life events and comorbidity). Secondly, setting general PROM-related goals is difficult because treatment may aim to improve HR-QoL in the case of impaired well-being caused by prolactinoma symptoms or DA side effects, whereas the aim may be stabilization of HR-QoL in the case of a preventive debulking. Moreover, a lack of reference values prevents meaningful interpretation of results. Thus, further research is required to determine how to incorporate PROMs in prolactinoma outcome sets.

Comparison of treatment outcomes and sharing experiences and data across PTCOEs is essential to improve prolactinoma care, considering it is a rare disease. The concepts postulated in this study may be used to determine suitable outcome parameters for the creation of an international database. Pituitary specialists should evaluate these outcome parameters to achieve expert consensus on the most important variables for such a database. Future studies should focus on the patient's perspective on treatment success, for instance using interviews. Moreover, the costs of outcome evaluations should be assessed (eg, by analyses using quality-adjusted life-years).

## Conclusion

Heterogeneity in reported outcome parameters with varying definitions hampers the comparison of treatment outcomes in prolactinomas. Using a standardized, objective, easily applicable, and clinically relevant outcome set enables comparison of outcomes across treatments and centers. This study provides an outcome set based on the VBHC principles, adaptable to various treatment goals. PROMs should be included as 1 of the core outcomes, yet further research is required to elucidate appropriate strategies to include them in objective treatment goals.

## Data Availability

The dataset analyzed during the current study is not publicly available but is available from the corresponding author on reasonable request.
